# A Study on User Recognition Using the Generated Synthetic Electrocardiogram Signal

**DOI:** 10.3390/s21051887

**Published:** 2021-03-08

**Authors:** Min-Gu Kim, Sung Bum Pan

**Affiliations:** IT Research Institute, Chosun University, Gwangju 61452, Republic of Korea; happy9433@gmail.com

**Keywords:** ECG, biometrics, user recognition, ACGAN, parallel ensemble networks

## Abstract

Electrocardiogram (ECG) signals are time series data that are acquired by time change. A problem with these signals is that comparison data that have the same size as the registration data must be acquired every time. A network model of an auxiliary classifier based generative adversarial neural network that is capable of generating synthetic ECG signals is proposed to resolve the data size inconsistency problem. After constructing comparison data with various combinations of the real and generated synthetic ECG signal cycles, a user recognition experiment was performed by applying them to an ensemble network of parallel structure. Recognition performance of 98.5% was demonstrated when five cycles of real ECG signals were used. Moreover, 98.7% and 97% accuracies were provided when the first cycle of synthetic ECG signals and the fourth cycle of real ECG signals were repetitively used as the last cycle, respectively, in addition to the four cycles of real ECG. When two cycles of synthetic ECG signals were used with three cycles of real ECG signals, 97.2% accuracy was shown. When the last third cycle was repeatedly used with the three cycles of real ECG signals, the accuracy was 96%, which was 1.2% lower than the performance obtained while using the synthetic ECG. Therefore, even if the size of the registration data and that of the comparison data are not consistent, the generated synthetic ECG signals can be applied to a real life environment, because a high recognition performance is demonstrated when they are applied to an ensemble network of parallel structure.

## 1. Introduction

Among the biometric signals investigated in studies of user recognition methods, electrocardiogram (ECG) signals are bio-signals that are produced autonomously and show unique characteristics of individuals according to such factors as the heart’s position, size, and structure, as well as age and gender [1]. However, the public database used in the existing research has a problem that does not consider reproducibility because the data acquired once is classified as learning data/validation data/experimental data for experimentation. Also, the databases used in the conventional user recognition research using ECG signals are composed of registration data and comparison data with the same size in the initial experimental environment setting [2]. However, ECG signals are time series data acquired by time change, and, if the comparison data are not of the same size as the registration data because of a lack of time for acquiring the comparison data, a problem of data size inconsistency occurs [3,4,5].

To solve this problem, when conventional data normalization methods such as data copy and interpolation method are applied to the ECG signal and converted into data of the same size, important characteristic information such as time information, amplitude, and interval of P, QRS, and T waves is lost. In addition, the heart rate and waveform of the ECG signal changes due to the individual’s physical activity, measurement time period, or mental influence, and accordingly, a section in which the waveform changes significantly and a section with relatively little change occurs.

**Figure 1:**
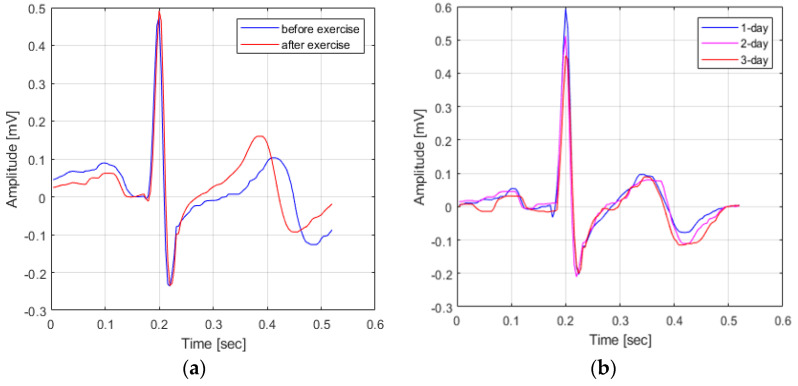
Variation in ECG signals based on waveform changes. (**a**) Variation in ECG signals based on a change in user state (**a**) and Variation in ECG signals change in time (**b**).

In fact, as shown in Figure 1, when comparing the waveform of the ECG signal in the pre-exercise state and the ECG signal in the post-exercise state, it can be seen that the P-peak point and the T-peak point occur closer to the QRS complex. In other words, if the ECG signal acquired in response to the user’s condition change is used as comparison data, not the ECG signal acquired in the same environment, the cause of user recognition performance deterioration occurs.

To solve this problem, studies have been conducted for various data schemes using neural networks. A generative adversarial network (GAN), which is the algorithm attracting the most attention in recent studies, is a model that generates data through adversarial learning of a generator and discriminator composed of two mutually different multilayer perceptrons. With a GAN, generation of data is simple, but it has the inherent problem of learning instability, because its approach in the learning process is a minimization problem [6].

In this study, synthetic data were generated through an auxiliary classifier GAN (ACGAN) using the class information as an auxiliary classifier. The structure of the ACGAN used in this study was designed to have mutually different convolution neural network (CNN) models for the generator and the discriminator. For the generation of one-cycle ECG signals, the input data used for the generator were the class information and the noise that had the same size as the cycle to be generated [7,8]. Because the real ECG signals and class information and the synthetic ECG signals and class information are divided, the structure of the discriminator model is designed as a CNN model that repeats the convolution operation of a structure that is not deep when compared with that of the generator model. The data are composed of various combinations of generated synthetic ECG signal cycles and real ECG signal cycles and applied to the user recognition.

This work is organized as follows: In Section 2, previous studies for generative adversarial-neural-network-based synthetic data generation are analyzed and conventional user recognition methods which use the ECG signal. In Section 3, the synthetic data generation model using the ACGAN proposed in this study and the ensemble network of parallel structure for user recognition are described. In Section 4, the similarity between the results of the real ECG signals and the generated synthetic ECG signals is analyzed, and the user recognition performance using the synthetic ECG signals is examined. Section 5 provides the conclusion of this work.

## 2. Related Works

### 2.1. Generated Synthetic Data Based on Deep Learning

The construction of large-scale data using personal biometric information is difficult in the field of medicine because of constraints, such as private information protection and acquisition cost; as a result, the problem of imbalanced big data arises. Imbalanced big data consist of majority classes containing larger data than other classes and minority classes containing smaller data than other classes. If a machine-learning model is learned using imbalanced big data, it is biased toward the majority classes, and, consequently, a problem occurs: low recognition performance is shown for the minority classes, or the possibility of recognizing newly input data as minority classes decreases.

**Figure 2:**
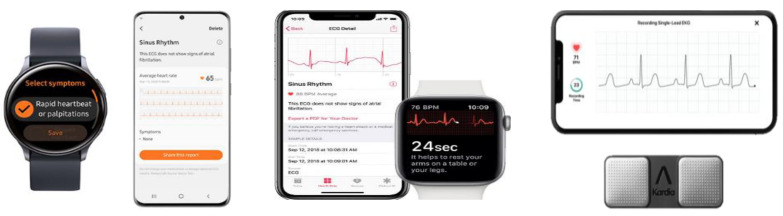
Wearable device based biosignal acquisition equipment.

In recent years, wearable device products like those shown in Figure 2 (Apple Watch, Galaxy Watch and Kardiamobile) have been commercialized, making it possible to acquire bio-signals in various environments. However, the number of bio-signal data is remarkably insufficient to be applied to a deep learning technology that exhibits high user recognition performance. In the existing research, a model-based synthetic data generation technology based on user-settable average heart rate, number of beats, sampling frequency, and waveform shape (P, Q, R, S and T timing, amplitude and duration) was researched. However, while the existing model-based synthetic data generation technology has the advantage of generating synthetic data with a small number of data, it has a disadvantage that it cannot be applied to data of a new waveform [9]. To resolve this problem, studies have been performed recently for various data generation methods using GANs. The GAN has shown excellent results in diverse areas, such as image generation, resolution improvement, and natural-language processing, as a method of a typical generation model using deep learning. AGAN consists of a generator model that generates data and a discriminator that classifies whether the data are real data or generated data. The main learning method is to use the adversarial structure of generator and discriminator to improve gradually the performance of each other. After finding the distribution of learning data, the GAN induces the generated data to follow that distribution, thereby generating data clearer than the variational auto-encoder. Using this characteristic, Christian et al. [10] proposed a super resolution GAN, which is a GAN-based high-resolution method. In the case of conventional methods using the loss function as mean squared error, a data-blurring phenomenon occurs, because the mean value of all possible solutions is estimated by data. However, the GAN can generate clearer data, because the learning is performed to be as close as possible to the learning data while maintaining individual attributes.

Hartmann et al. [11] used a Wasserstein GAN to apply various upsampling and downsampling methods to a method of generating ECG signals, which are time series data. The Wasserstein GAN improved the instable learning of a conventional GAN by using the concept of the Wasserstein Distance in the loss function instead of using conventional loss functions, such as Kullback-Leibler Divergence or Jensen-Shannon Divergence. Wasserstein distance is an indicator measuring the distance between two probability distributions and refers to the minimal cost used to let a certain probability distribution shape have a different probability distribution shape. Furthermore, the up-sampling methods used in the discriminator model were nearest-neighbor, linear, and cubic interpolation. However, for the down-sampling used in the generator model, the impact on data generation was investigated by applying average pooling. Table 1 shows the structures of generator and discriminator used in Wasserstein GAN.

Tomer et al. [12] proposed personalized generative adversarial networks to generate the P, QRS, and T wave features of ECG signals similar to those of real ECG signals by patients using the MIT-BIH arrhythmia data. For the input data of the generator model, 100 arbitrary samples in the Gaussian distribution noise were used, and batch normalization and rectified linear unit (ReLU), which is an activation function, were applied to the deconvolution layer. Instead of one cycle, three cycles of ECG signals were generated, and the middle cycle was used as input data of the discriminator. The remaining two cycles were used as input data of mean squared error, which is a loss function used in the discriminator model, after detecting the P, QRS, and T waves. In the results of applying the ECG signals generated for each patient to the long short-term memory model to check the classification performance, 0.95 area-under-curve performance was shown, confirming that the classification performance improved when the learning was performed by adding the ECG signals generated for each patient.

### 2.2. Deep Learning Networks Design Using ECG Signals

A variety of studies have been performed on personal recognition using ECG signals based on deep learning. As a conventional method of applying the ECG signal to deep learning, Jun projected a one-dimensional ECG signal into a two-dimensional space to solve the problem of extracting various sampling rates and amplitudes depending on the acquisition equipment and to increase the limited ECG signal. The result of 99.05% recognition is shown by applying the 2D transformed data to the CNN. Compared to general 2D images, 2D ECG images showed simple patterns and showed high recognition performance without deep network design.

Zhai et al. [13] transformed ECG signals into 2D images, and applied these to the CNN, which subsequently showed an accuracy of 98.6% and 97.5% for the evident waveform detection. Ubeyli [14] proposed a method of detecting arrhythmia with the RNN, using principal component analysis based feature extraction. Experimental results showed that this network has an average accuracy of 98.06% for four different types of arrhythmia. Zubair et al. [15] designed a nine layer CNN with an accuracy of 92.7%. Acharya et al. [16] designed a nine layer CNN with an accuracy of 94.03% and 93.49% for waveforms before and after noise remove, respectively.

**Figure 3:**
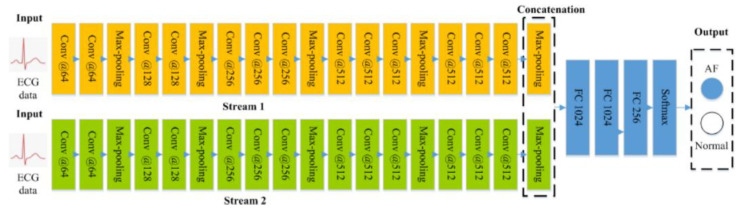
Example of two stream based convolutional neural networks.

Kiranyaz et al. [17] applied a 1D CNN to ECG arrhythmia detection. Unlike the method of applying the CNN to 2D ECG images, Kiranyaz’s method showed excellent performance results by applying the CNN to 1D ECG signals. Rajpurkar et al. [18] proposed a 1D CNN model that used a deeper network and more numerous data than Kiranyaz’s CNN model. However, detection performance was low in spite of the use of more ECG data. Fan et al. [19] proposed a two layer multi scale CNN model (shown in Figure 3) to detect normal ECG signals and arrhythmia ECG signals. Its structure was designed with different filter sizes for each layer, to detect features of different data scales, and they applied a database of the ECG signal sampled at 20 s intervals. Experimental results showed that the multi scale CNN model they proposed achieved a detection result of 98.13%, which was an highly improvement upon the results of 89.58% and 98.03% achieved when a single network and the VGG network were applied, respectively.

Liu et al. [20] proposed an ensemble network that combines multi stream CNNs and a single RNN to classify myocardial infarction signals using 12 lead ECG signals. They designed multiple independent CNNs to receive input different signals from the 12 leads ECG signals. The feature data output from each network were used as the input data of the single RNN. They also solved the overfitting problem between the multi stream CNNs and the single RNN by applying the lead random mask (LRM). Lead random mask solves a generalization problem that occurs when processing large amounts of data in training, using randomly selected data in the same way as Dropout. Experimental results showed that the detection performance for myocardial infarction and normal signals achieved a classification rate of 99.9%, and the recognition rate of experimental subjects was 93.08%. Oh et al. [21] proposed an ensemble network model using a single CNN and single RNN to diagnose five types of arrhythmia. The single CNN was designed to enable a classifier to extract spatial features, and arrhythmia signals were detected by applying the detected features to the single RNN, which receives data according to temporal information. The results of applying public databases of arrhythmia signals showed a high detection rate of 98.1%. As described above, previous studies on user recognition using ECG signals have recently gained attention as a next generation user recognition method that can replace conventional recognition methods effectively, owing to their high accuracy.

## 3. Proposed User Recognition Using Synthetic ECG Signal

### 3.1. Synthetic ECG Generation of GAN Using Auxiliary Classifier

The ACGAN is a model that generates data by using input class information, and it discriminates the classes of generated data through the auxiliary classifier. While the conventional discriminator performs learning to discriminate whether data are real data or generated synthetic data, the ACGAN performs learning to classify the data classes as well. The generator generates data not to deceive the discriminator, but to produce accurate classification results for the generated data. The data generated through such a learning process can obtain similar results to the real data. Furthermore, data of a certain class can be generated using the class information.

**Figure 4:**
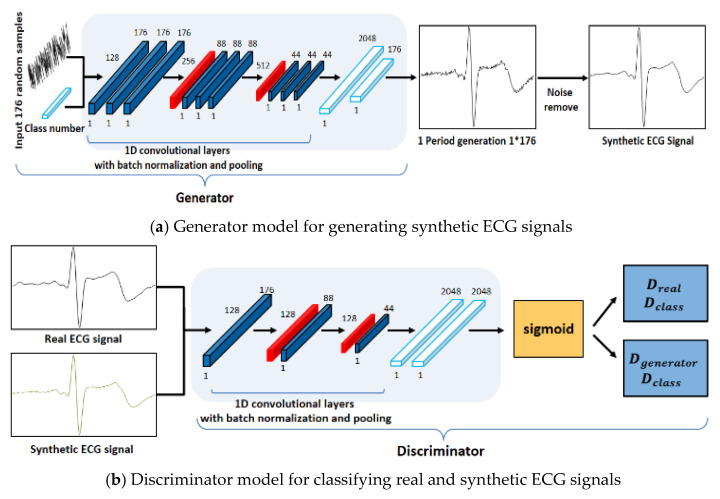
Proposed generator and discriminator network structures.

The structure of the ACGAN used in this study was designed to ensure that the generator and the discriminator have mutually different CNN models, as shown in Figure 4 [22]. To generate one-cycle ECG signals, the generator use the following input data: the class information and noise with the same size as the cycle to be generated. Data are generated in the generator model using a one-dimensional convolution operation and pooling operation repetitively and these data are used as the input data of the discriminator model along with the class information. As the number of layers increased through the repeated experiments, the generator model could not generate data similar to the real ECG signals; on the contrary, the values of generated synthetic ECG signals diverged because of the data loss in the learning process.

Therefore, the generator model was designed with nine convolutional layers, two pooling layers, and a fully connected layer. The learning is performed for the discriminator model in a direction of classifying the signals and classes of the real ECG and the synthetic ECG input in the discriminator model. The structure of the discriminator model is designed with a CNN model that repeats an operation of convolution and has a shallower structure than the generator model, because it classifies the real ECG signals and class information and the synthetic ECG signals and class information. Accordingly, the generator and discriminator models generated synthetic ECG signals similar to the real ECG data through the repeated learnings.

### 3.2. Ensemble Networks Design of Parallel Structure

**Figure 5:**
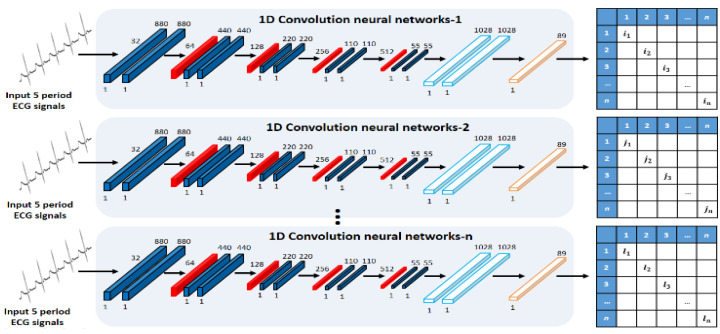
Single CNN of parallel structure.

**Figure 6:**
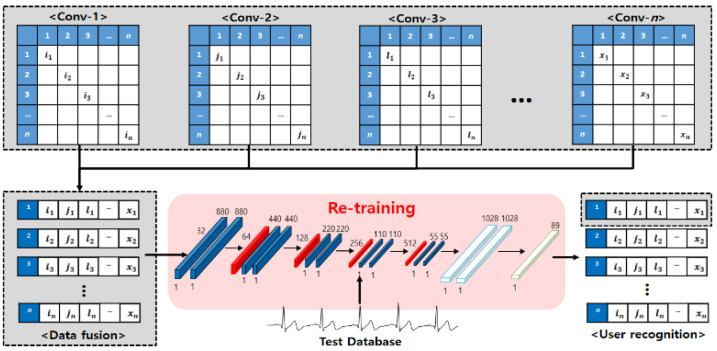
Single network for retraining.

An ensemble network structure of parallel structure was designed using acquired ECG signals by user status change, as shown in Figure 5 and Figure 6. First, the real data acquired by the user status and environment changes constitute the registration database, and they are used as input data in the 1D single CNN of parallel structure. The 1D single network structure uses alternately a convolution layer that detects unique features of ECG signals and converts them into a feature map through convolution operation, a pooling layer that reduces the data size, and a dense layer that sets the inputs and size. The role of the pooling layer is to reduce the volume of calculation by reducing the size of data output from the convolution layer, and to facilitate extraction of features having robust properties [23].

The average pooling produces an effect of reducing the features of robust properties output from the convolution layer (down-scale weighting) when “0” is output many times for the operation result by ReLU, an activation function. Therefore, max-pooling, in which the maximum value is selected in the window, is used. The ensemble method improves the performance through combinations of mutually different models, and each 1D single network is composed with different parameters to detect different features. The learning is set to perform 500 and 750 times repeatedly, and the batch size, which is the number of data used in the learning each time, is set to 256 and 512. The dropout that reduces the computation time and amount by omitting a part of the network is set to between 50~70%. For the learning rate, 0.001 is applied, which is often used in general.

Next, the ECG signals of output results from each network are used as registration data for retraining by combining them into one database. However, when the ECG signals output from each network are all used as registration data, even the results of low recognition rates are used as registration data because of the parameters and incorrect network design; consequently, there is a problem that the recognition performance declines. Therefore, not all output data are used, and by combining the result data of the top-three networks showing excellent performance, the registration data are composed. Finally, user recognition is performed for the recomposed registration data by relearning the time-independent comparison data in the single CNN.

## 4. Experimental Results

The type of noise in the ECG signal is a power line interference arising from the device used for acquiring the ECG signal, motion artifacts caused by subjects’ movement, muscle contractions by irregular muscle activity, and baseline drift by breathing [24]. This noise can provide false information and degrade user recognition performance, so the noise removed process is essential [25]. First, degree of reduction for each frequency depends on the design and parameters of the filter. By using the high pass filter with a cutoff frequency of 0.5 Hz, we remove baseline drift in the low frequency band. Next, to remove power line interference, we applied the notch filter for the 60 Hz band. Lastly, we perform R-peak wave detection using the Pan and Tompkins method.

**Figure 7:**
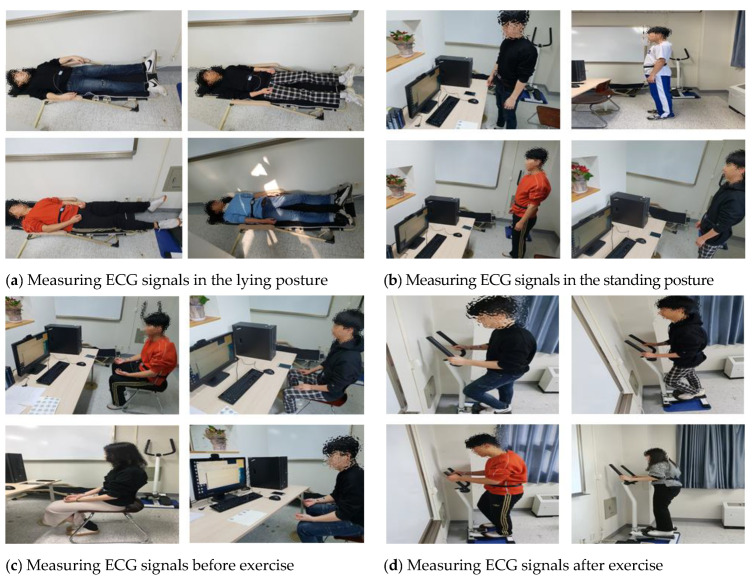
Definition of user states for changing ECG signals.

ECG signals were acquired from 89 adults of various age groups from their 20s to 50s to analyze the ECG signal changes and apply them to user recognition. The equipment used for measurement was an MP160 model (BIOPAC Systems, CA, USA) and lead-1 ECG signals were acquired by using wet electrodes. Measurements were taken over the course of one year, in order to acquire time independent ECG signals, and the ECG signals were measured by defining the following four environment as ones that could change ECG signals in real environment as show in Figure 7 [26]. Depending on the subject’s schedule, ECG signals were measured three times at a 2000 Hz sampling rate across different days.

- Lying down: acquiring signals for 1-min in the lying posture after rest state
- Standing: acquiring signals for 1-min in the standing posture after rest state
- Before exercise: acquiring signals for 1-min in the sitting position before exercise after rest state
- After exercise: acquiring signals for 1-min while maintaining the heart rate above 120 through the stepper exercise equipment after exercise

The similarity between the acquired ECG signals and the generated synthetic ECG signals was measured and checked using the cosine similarity and cross correlation [27,28]. First, the cosine similarity test results were examined to investigate the directional similarity between the real and synthetic ECG signals. The cosine similarity is in the range of −1 to 1. As it approaches 1, it indicates a high similarity between the two signals, and, as it approaches -1, it indicates a signal of different waveform. Table 2 exhibits the cosine similarity measurement results by experimentee and shows a minimum similarity of 0.974 and a maximum similarity of 0.998, with a mean cosine similarity of 0.991.

**Figure 8:**
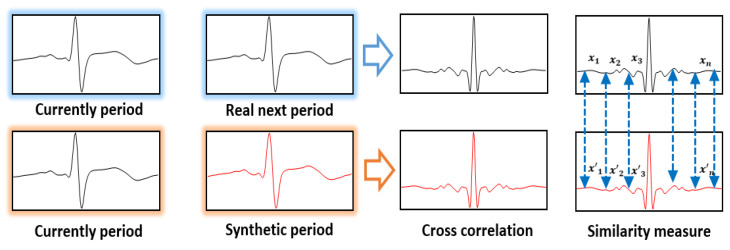
Similarity measurement method using euclidean distance on the basis of cross correlation.

Next, the similarity results were checked using the cross correlation quantified through the correlation analysis to investigate how similar two different signals are in the signal-processing area. After overlapping the cross-correlation waveforms of the current ECG signal cycle and the synthetically generated ECG signal cycle on the cross-correlation waveforms output from the current ECG signal cycle and the next ECG signal cycle, the similarity between the two signals was expressed numerically by using the Euclidean distance. Figure 8 shows a method of measuring similarity using Euclidean distance based on cross correlation.

As the Euclidean distance of two cross correlations approaches 0, it indicates the generation of synthetic ECG signals similar to the real ECG signals of the same experimentee; as it moves away from 0, it indicates a cross-correlation result of real ECG signals and synthetic signals between different experimentees.

Table 3 is the similarity measurement results using Euclidean distance based on the cross correlation by experimentee, and it shows the similarities from 0.136 minimum to 0.364 maximum, with the mean Euclidean distance showing a similarity result of 0.25. In the results of using the network proposed, the directivity features are very similar to the real ECG signals, and the synthetic ECG signal generation results have different correlation waveforms between the experimentees. This means that the generated synthetic ECG signals were similar to the real ECG signals, and even if the registration and comparison data do not have the same size, the data size inconsistency problem was solved through synthetic data generation.

Because ECG signals of certain cycles are difficult to acquire in a real-life environment, the comparison data were constructed with various combinations of the real ECG signal cycles and the generated synthetic ECG signal cycles, as shown in Table 4. Then, they were applied to the ensemble network of parallel structure. The number of data used in the experiment was as follows: 53,400 data were used for the training data through the data expansion method with 600 data per person acquired from 89 experimentees over three times, and 17,800 data were used for the validation and comparison data.

**Figure 9:**
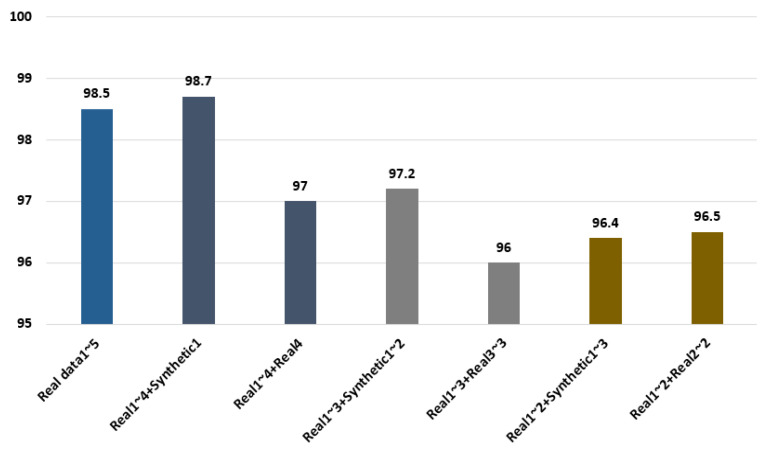
Comparison of recognition performance using the real and synthetic ECG signals.

In the experimental results, 98.5% recognition performance was shown when the five cycles of real ECG signals were used, as shown in Figure 9, and 98.7% and 97% accuracies were shown, respectively, as results of repeatedly using the one cycle of synthetic ECG signals and the fourth cycle for the last cycle with the four cycles of real ECG signals. A 97.2% accuracy was shown when two cycles of synthetic ECG signals were used with three cycles of real ECG signals. Moreover, when the last third cycle was repeatedly used with the three cycles of real ECG signals, 96% accuracy was demonstrated, which was 1.2% lower than the performance obtained using the synthetic ECG signals. Therefore, the generated synthetic ECG signals are similar to the real ECG signals. Furthermore, the data composed by combining the synthetic ECG signals demonstrated superior recognition performance compared with the data using the real ECG signals repetitively. Therefore, a high user recognition result is confirmed through the ensemble network of parallel structure using the synthetic ECG signals.

In addition, the performance of the latest technology was compared using the MIT-BIH database, an open database. As shown in Table 5, the performance of Kim [22] was 99.6%, which was higher or similar to the previous studies. This is the result of applying a public database measured once in the same state as training, verification, and test data to minimize the effect of heart rate and waveform changes. In other words, it can be interpreted as a result of recognizing the environment at the time the data was acquired, not the result of recognizing the experimenter’s unique characteristics.

## 5. Conclusions

In conventional user recognition studies using ECG signals, experiments are performed by constructing comparison data with the same size as the registration data in the initial experimental environment. However, when the size is not identical to that of the registration data because of a lack of comparison data acquisition time, they cannot be applied to user recognition because of the data size inconsistency problem. To resolve this problem, an ACGAN network model was proposed. In this study, the similarity was measured using the cosine similarity and the cross correlation to evaluate the generated synthetic ECG signals. Furthermore, after constructing comparison data with various combinations of the real ECG signal cycles and the generated synthetic ECG signal cycles, the user recognition experiment was conducted by applying them to an ensemble network of parallel structure.

In the experimental results, the proposed ACGAN confirmed the data generation of various features, and even when the size was not consistent between the registration and comparison data, the data size inconsistency problem was solved through generation of synthetic data. Furthermore, because ECG signals of certain cycles are difficult to acquire in a real-life environment, the comparison data were constructed with various combinations of the generated synthetic ECG signal cycles and the real ECG signal cycles, in which heart rate and waveform changes occurred in real life according to the user status. Then, the user recognition experiment was conducted by applying them to the ensemble network of parallel structure.

In the experimental results, the recognition performance obtained when the synthetic ECG signals were used was higher than that obtained when the real ECG signals were repeatedly used. In other words, high recognition performance was shown when the generated synthetic ECG signals were applied to an ensemble network of parallel structure according to the user status changes, even if the size was not consistent between the registration and comparison data. Therefore, the applicability of the proposed model in real-life environments was confirmed. The study was conducted focusing on generation of synthetic ECG signals similar to real ECG signals. At present, synthetic ECG signals similar to real ECG signals can be generated only if the learning process is undergone for the generation of synthetic ECG signals. Therefore, a study for real-time generation of ECG signals using user information learned in advance has been planned.

## Figures and Tables

**Table 1:** Structures of generator and discriminator models.

Generator			Critic		
Layers	Act/Norm	Output Shape	Layers	Act/Norm	Output Shape
Latent	-	200×1	Input	-	1×768
Linear	LReLU	50×12	Conv 1	LReLU	50×768
Upsample	-	50×24	Conv 9	LReLU	50×768
Conv 9	LReLU/PN	50×24	Conv 9	LReLU	50×768
Conv 9	LReLU/PN	50×24	Downsample	-	50×384
Upsample	-	50×48	Conv 9	LReLU	50×384
Conv 9	LReLU/PN	50×48	Conv 9	LReLU	50×384
Conv 9	LReLU/PN	50×48	Downsample	-	50×1 92
Upsample	-	50×96	Conv 9	LReLU	50×1 92
Conv 9	LReLU/PN	50×96	Conv 9	LReLU	50×1 92
Conv 9	LReLU/PN	50×96	Downsample	-	50×96
Upsample	-	50×192	Conv 9	LReLU	50× 96
Conv 9	LReLU/PN	50×192	Conv 9	LReLU	50×96
Conv 9	LReLU/PN	50× 192	Downsample	-	50×48
Upsample	-	50×384	Conv 9	LReLU	50×48
Conv 9	LReLU/PN	50×384	Conv 9	LReLU	50×48
Conv 9	LReLU/PN	50× 384	Downsample	-	50×24
Upsample	-	50×768	Conv 9	-	50×24
Conv 9	LReLU/PN	50×768	Conv 9	LReLU	50×24
Conv 9	LReLU/PN	50×768	Downsample	-	50×1 2
Conv 1	-	1×768	Linear	-	1×1

**Table 2:** Cosine similarity measurement results by experimentee.

**Class**	**1**	**2**	**3**	**4**	5	**6**	**7**	**8**	**9**
**Result**	0.998	0.996	0.996	0.995	0.996	0.991	0.99	0.997	0.994
**Class**	**10**	**11**	**12**	**13**	**14**	**15**	**16**	**17**	**18**
**Result**	0.99	0.989	0.988	0.991	0.979	0.991	0.974	0.995	0.994
**Class**	**19**	**20**	**21**	**22**	**23**	**24**	**25**	**26**	**27**
**Result**	0.993	0.991	0.992	0.99	0.989	0.994	0.992	0.994	0.98
**Class**	**28**	**29**	**30**	**31**	**32**	**33**	**34**	**35**	**36**
**Result**	0.995	0.996	0.993	0.99	0.979	0.996	0.989	0.996	0.995
**Class**	**37**	**38**	**39**	**40**	**41**	**42**	**43**	**44**	**45**
**Result**	0.993	0.987	0.99	0.989	0.996	0.995	0.995	0.975	0.987
**Class**	**46**	**47**	**48**	**49**	**50**	**51**	**52**	**53**	**54**
**Result**	0.988	0.984	0.93	0.994	0.994	0.994	0.985	0.986	0.997
**Class**	**55**	**56**	**57**	**58**	**59**	**60**	**61**	**62**	**63**
**Result**	0.995	0.998	0.978	0.998	0.996	0.98	0.996	0.995	0.993
**Class**	**64**	**65**	**66**	**67**	**68**	**69**	**70**	**71**	**72**
**Result**	0.982	0.996	0.996	0.992	0.995	0.993	0.988	0.995	0.995
**Class**	**73**	**74**	**75**	**76**	**77**	**78**	**79**	**80**	**81**
**Result**	0.992	0.995	0.994	0.99	0.992	0.987	0.994	0.996	0.989
**Class**	**82**	**83**	**84**	**85**	**86**	**87**	**88**	**89**	**AVG**
**Result**	0.994	0.996	0.996	0.989	0.995	0.992	0.995	0.998	0.991

**Table 3:** Similarity measurement results using euclidean distance on the basis of cross correlation.

**Class**	**1**	**2**	**3**	**4**	**5**	**6**	**7**	**8**	**9**
**Result**	0.264	0.352	0.166	0.247	0.212	0.31	0.178	0.242	0.36
**Class**	**10**	**11**	**12**	**13**	**14**	**15**	**16**	**17**	**18**
**Result**	0.136	0.254	0.223	0.176	0.198	0.261	0.36	0.163	0.227
**Class**	**19**	**20**	**21**	**22**	**23**	**24**	**25**	**26**	**27**
**Result**	0.325	0.298	0.271	0.35	0.221	0.259	0.197	0.362	0.296
**Class**	**28**	**29**	**30**	**31**	**32**	**33**	**34**	**35**	**36**
**Result**	0.27	0.344	0.168	0.19	0.224	0.314	0.356	0.172	0.34
**Class**	**37**	**38**	**39**	**40**	**41**	**42**	**43**	**44**	**45**
**Result**	0.198	0.21	0.332	0.281	0.26	0.314	0.171	0.364	0.218
**Class**	**46**	**47**	**48**	**49**	**50**	**51**	**52**	**53**	**54**
**Result**	0.323	0.167	0.229	0.284	0.341	0.217	0.29	0.192	0.23
**Class**	**55**	**56**	**57**	**58**	**59**	**60**	**61**	**62**	**63**
**Result**	0.276	0.139	0.224	0.261	0.25	0.317	0.163	0.183	0.266
**Class**	**64**	**65**	**66**	**67**	**68**	**69**	**70**	**71**	**72**
**Result**	0.189	0.16	0.335	0.238	0.162	0.281	0.318	0.29	0.314
**Class**	**73**	**74**	**75**	**76**	**77**	**78**	**79**	**80**	**81**
**Result**	0.195	0.263	0.225	0.242	0.22	0.196	0.329	0.214	0.31
**Class**	**82**	**83**	**84**	**85**	**86**	**87**	**88**	**89**	**AVG**
**Result**	0.324	0.161	0.243	0.324	0.212	0.194	0.22	0.139	0.25

**Table 4:** Experimental database for comparing user recognition performance.

	Test Data Set				
**Real1~5**	**Real1**	**Real2**	**Real3**	Real4	Real5
**Real1~4+Synthetic1**	Real1	Real2	Real3	Real4	**Synthetic1**
**Real1~4+Real4**	Real1	Real2	Real3	Real4	Real4
**Real1~3+Synthetic1~2**	Real1	Real2	Real3	**Synthetic1**	**Synthetic2**
**Real1~3+Real3~3**	Real1	Real2	Real3	Real3	Real3
**Real1~2+Synthetic1~3**	Real1	Real2	**Synthetic1**	**Synthetic2**	**Synthetic3**
**Real1~2+Real2~2**	Real1	Real2	Real2	Real2	Real2

**Table 5:** Comparison of recognition performance with previous studied using MIT-BIH data.

Classifier	Work	Database	Test Set	Accuracy	Specificity	Sensitivity
1D Ensemble Networks	Kim [22]	MIT-BIH database	1692	99.6%	0.99	0.99
2D CNN	Jun et al. [29]		100,000	99%	0.99	0.97
	Abdeldayem et al. [30]		250	98.8%	-	-
1D CNN	Zhang et al. [31]		250	91.1%	-	-
MLP	Sidek et al. [32]		-	94.4%	0.99	0.94
RBF				96.2%	0.99	0.96
KNN				97.9%	0.99	0.97

## Data Availability

Not applicable.
