# Correction: Kim, M.-G.; Pan, S.B. A Study on User Recognition Using the Generated Synthetic Electrocardiogram Signal. *Sensors* 2021, *21*, 1887

**DOI:** 10.3390/s24144652

**Published:** 2024-07-18

**Authors:** Min-Gu Kim, Sung Bum Pan

**Affiliations:** IT Research Institute, Chosun University, Gwangju 61452, Republic of Korea; happy9433@gmail.com

In the original publication [1], the “Acknowledgments” section was not included. This section should include the following paragraph:

The article was written based on the first author’s doctoral dissertation [22].

## Front Matter Correction

The email address of first author has been changed from happy9433@chosun.ac.kr to happy9433@gmail.com.

## Text Correction

In the second paragraph of Section 3.1, the sentence “The structure of the ACGAN used in this study was designed to ensure that the generator and the discriminator have mutually different CNN models, as shown in Figure 4” has been updated to “The structure of the ACGAN used in this study was designed to ensure that the generator and the discriminator have mutually different CNN models, as shown in Figure 4 [22].”

In the last paragraph of Section 4, the sentence “As shown in Table 5, the performance of the proposed method was 99.6%, which was higher or similar to the previous studies.” has been updated to “As shown in Table 5, the performance of Kim [22] was 99.6%, which was higher or similar to the previous studies.”

## Table Correction

Table 5 is updated as appear below:

## References Correction

The newly added references appear below:

22. Kim, M.G. A Study on User Recognition System Based on Ensemble Convolutional Neural Networks Using Synthetic Electrocar-Diogram Generation. Doctoral Dissertation, Department of Control and Instrumentation Engineering, Chosun University, Gwangju, Republic of Korea, 2019.

The authors apologize for any convenience caused and state that the scientific conclusions are unaffected. With this correction, the order of some references has been adjusted accordingly. This correction was approved by the Academic Editor. The original article has been updated.

## Figures and Tables

**Table 5:** Comparison of recognition performance with previous studied using MIT-BIH data.

Classifier	Work	Database	Test Set	Accuracy	Specificity	Sensitivity
1D Ensemble Networks	Kim [22]	MIT-BIH database	1692	99.6%	0.99	0.99
2D CNN	Jun et al. [29]		100,000	99%	0.99	0.97
	Abdeldayem et al. [30]		250	98.8%	-	-
1D CNN	Zhang et al. [31]		250	91.1%	-	-
MLP	Sidek et al. [32]		-	94.4%	0.99	0.94
RBF				96.2%	0.99	0.96
KNN				97.9%	0.99	0.97
